# Characterization of the Primo-Vascular System in the Abdominal Cavity of Lung Cancer Mouse Model and Its Differences from the Lymphatic System

**DOI:** 10.1371/journal.pone.0009940

**Published:** 2010-04-01

**Authors:** Jung Sun Yoo, M. Hossein Ayati, Hong Bae Kim, Wei-bo Zhang, Kwang-Sup Soh

**Affiliations:** 1 Department of Physics and Astronomy, Seoul National University, Seoul, Korea; 2 School of Acupuncture, Beijing University of Chinese Medicine, Beijing, China; 3 Institute of Acupuncture and Moxibustion, China Academy of Chinese Medical Science, Beijing, China; Health Canada, Canada

## Abstract

Cancer growth and dissemination have been extensively studied for a long time. Nevertheless, many new observations on anatomy and histopathology of cancer events are still reported such as formation of a vasculogenic-like network inside aggressive tumors. In this research, new kinds of micro-conduits, named primo-vessels, were found inside the abdominal cavity of NCI-H460 lung cancer murine xenograft models. These vascular threads were largely distributed on the surfaces of various organs and were often connected to peritoneal tumor nodules. Histological and immunofluorescent investigations showed that the primo-vessels had characteristic features that were distinctively different from those of similar-looking lymphatic vessels. They had multiple channels surrounded with loose collageneous matrices, which is in contrast to the single-channel structure of other vascular systems. The rod-shaped nuclei aligned longitudinally along the channels were assumed to be the endothelial cells of the primo-vessels, but LYVE-1, a specific marker of lymphatics, was not expressed, which indicates a clear difference from lymphatic endothelial cells. Taken together these findings on and characterization of the novel threadlike vascular structures in cancer models may have important implications for cancer prognosis and for therapy.

## Introduction

Primo-vessels (Bonghan ducts) are new kinds of micro-conduits and are assumed to be the mechanistic underpinning of acupuncture meridians in traditional oriental medicine. In the early 1960's, Bong-Han Kim of North Korea claimed to have discovered these ducts, named after him, which formed a novel circulatory system throughout an animal's body [1]. He did not, however, disclose his method of observing the system in detail, so until recently, no exact confirmation of his work was ever accomplished despite many attempts in China, Japan, and Russia, except, for a partial reproduction by a Japanese anatomist, Fujiwara [2].

Observations of primo-vessels in rabbits, rats, and mice were recently made by applying modern bio-imaging techniques [3], which has not only confirmed Kim's claims but also provided new findings and information, such as *in vivo* staining of the primo-vessels with Trypan blue [4] and the existence of primo-vessels inside fat tissues [5] and on the surfaces of subcutaneous tumor tissues [6]. Furthermore, some evidence for a fluid-conducting function of the primo-vascular system has been provided by measurements of the flow speed [7] and the fluid viscosity [8].

In the present work, we observed primo-vessels on the surfaces of abdominal organs and around peritoneal tumor nodules in lung cancer mouse models. To elucidate the distribution patterns of the primo-vessels in the context of cancer biology, we used two sets of cancer models, i.e., subcutaneous (s.c.) xenograft model and the intraperitoneal (i.p.) experimental metastasis model using administration of NCI-H460 human lung cancer cells [9], [10]. Trypan blue staining under *in vivo* and *in situ* conditions [4], [11] was applied to reveal the extent of the primo-vessels in tumor-bearing mice, which was possible due to the dye being preferentially absorbed into the loose structure of primo-vessels compare to other abdominal tissues.

We also performed histological and immunofluorescent investigations to distinguish the primo-vessels from similar-looking lymphatic vessels. The distinction between primo-vessels and lymphatic vessels has been an impending question because of the similarities of these morphological characteristics. Although this problem was partially resolved by using conventional histology and electron microscopy [12], [13], a more convincing examination, such as immunohistochemistry with a reliable molecular marker of lymphatic endothelial cells, LYVE-1 [14], [15], [16], had not been tried until this research. In which we performed immunostaining with LYVE-1, in addition to using various conventional histological examinations, to show the distinction between primo-vessels and lymphatic vessels.

## Results

### Intraoperative Visualization of Primo-vessels in s.c. Xenografts

**Figure 1:**
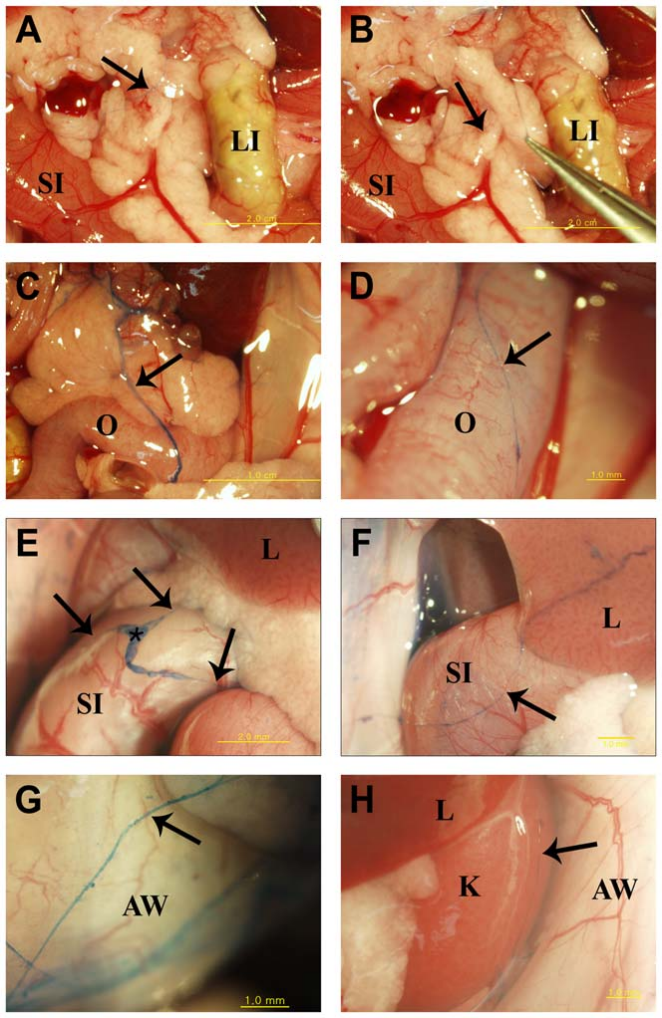
Intraoperative visualization of the primo-vascular system in abdominal cavity of s.c. lung cancer mouse models. *In situ* trypan blue spreading revealed semitransparent primo-vessels (arrows) which are distributed on the surfaces of various organs due to higher infiltration of the dye into loose extracellular structure of primo-vessels compare to other tissues. Straight thin primo-vessels were often visible, but sometimes many forked branches with a junction (an asterisk) were also found. Note that primo-vessel is liftable by forceps in contrast to lymphatic vessels (B). LI; large intestine, SI; small intestine, O; oviduct, L; liver, K; kidney, AW; abdominal wall.

To visualize primo-vessels in s.c. mouse xenografts, we performed careful abdominal surgery two to eight weeks after inoculation of 1×10^7^ NCI-H460 human lung cancer cells. Figure 1 presents anatomical images of the primo-vascular system, stained with trypan blue (blue color), inside the abdomens of s.c. tumor-bearing mice. *In situ* trypan blue spreading under live conditions revealed semitransparent primo-vessels (arrows) in fatty tissues (Figs. 1A & 1B) or around gonadal organs (Figs. 1C & 1D) or on the surfaces of various organs, including the small intestine, the kidney, and the liver (Figs. 1E, 1F & 1H), or on the abdominal wall (Fig. 1G). Other known structures, such as lymphatic vessels, adipose tissue, blood vessels, and nerves, were not *in vivo* stained with trypan blue in our several attempts. Straight, thin primo-vessels were often visible, but sometimes many forked branches with a junction (an asterisk) were also found. We could lift long and elastic primo-vessels with forceps whereas lymphatic vessels are commonly fixed inside tissues (Fig. 1B).

### Intraoperative Visualization of Primo-vessels in i.p. Xenografts

**Figure 2:**
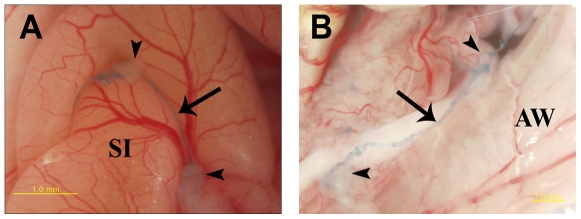
Intraoperative visualization of the primo-vascular system attached to tumor nodules in i.p. lung cancer mouse models. Primo-vessels (arrows) stained with trypan blue mainly located around tumor nodules (arrowheads) which were visible after 4 weeks of i.p. injection with NCI-H460 cells. Especially some primo-vessels showed distribution patterns either going inside or surrounding tumor nodules. SI; small intestine, AW; abdominal wall.

A similar intraoperative imaging was performed with i.p. mouse xenografts. Figure 2 depicts the primo-vascular system attached to intraperitoneal tumor nodules. The primo-vessels (arrows), stained with trypan blue, were mainly located around tumor nodules (arrowheads) that had formed at 4 weeks after i.p. injection with 1×10^7^ NCI-H460 cells. Especially, some primo-vessels showed distribution patterns either going inside or around tumor nodules and are likely to be new growths of primo-vessels driven by the needs of the tumor.

### Histological Characterization of Primo-vessels with H&E and MT Staining

**Figure 3:**
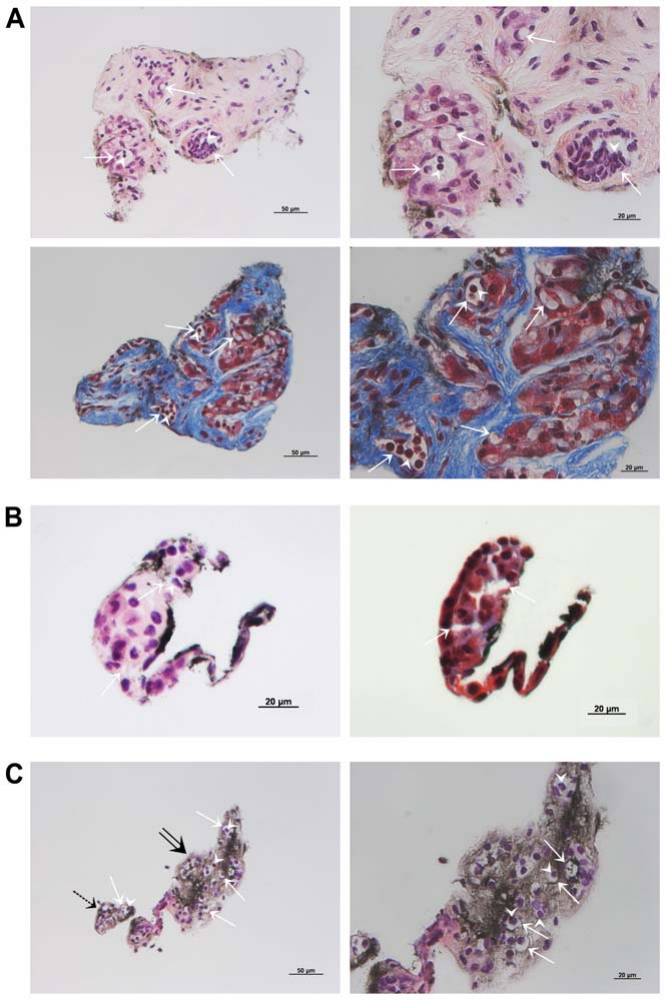
Histological examination of the primo-vascular system in lung cancer xenografts. (A) Shown are a representative hematoxylin and eosin (H&E)-stained primo-node cross-section (top) and a Masson's trichrome (MT)-stained adjacent-section (bottom). Right figures are enlarged images of left figures. (B) Depicted are primo-vessel cross-sections which were stained with H&E (left) and MT (right). (C) H&E staining of an oblique section containing both a primo-node (a double arrow) and a primo-vessel (a dotted arrow). An image of the primo-node was magnified in the right figure. There are multi lumens (arrows) with floating cells (arrowheads) in all samples which are distinct from a single lumen structure of lymph or blood vessels. Furthermore, MT staining revealed primo-vascular system consists of loose collagen fibers (blue color) surrounding lumens.

A further characterization to find a distinctive morphology for the primo-vessels was performed by using a histopatholgic examination. Figure 3 displays typical primo-nodes and primo-vessels obtained from lung cancer xenografts. Hematoxylin and eosin (H&E) and Masson's trichrome (MT) staining revealed multiple lumens (arrows) with floating cells (arrowheads) in all samples, and this multi-lumen structure is distinct from the single-lumen structure of lymph or blood vessels. Furthermore, MT staining revealed a primo-vascular system consisting of loose collagen fibers (blue color) surrounding the lumens. This result shows that primo-vessels cannot be mistaken for coagulated fibrins or other artifacts.

### Immunostaining with a Lymphatic Marker of Lymphatics, LYVE-1

**Figure 4:**
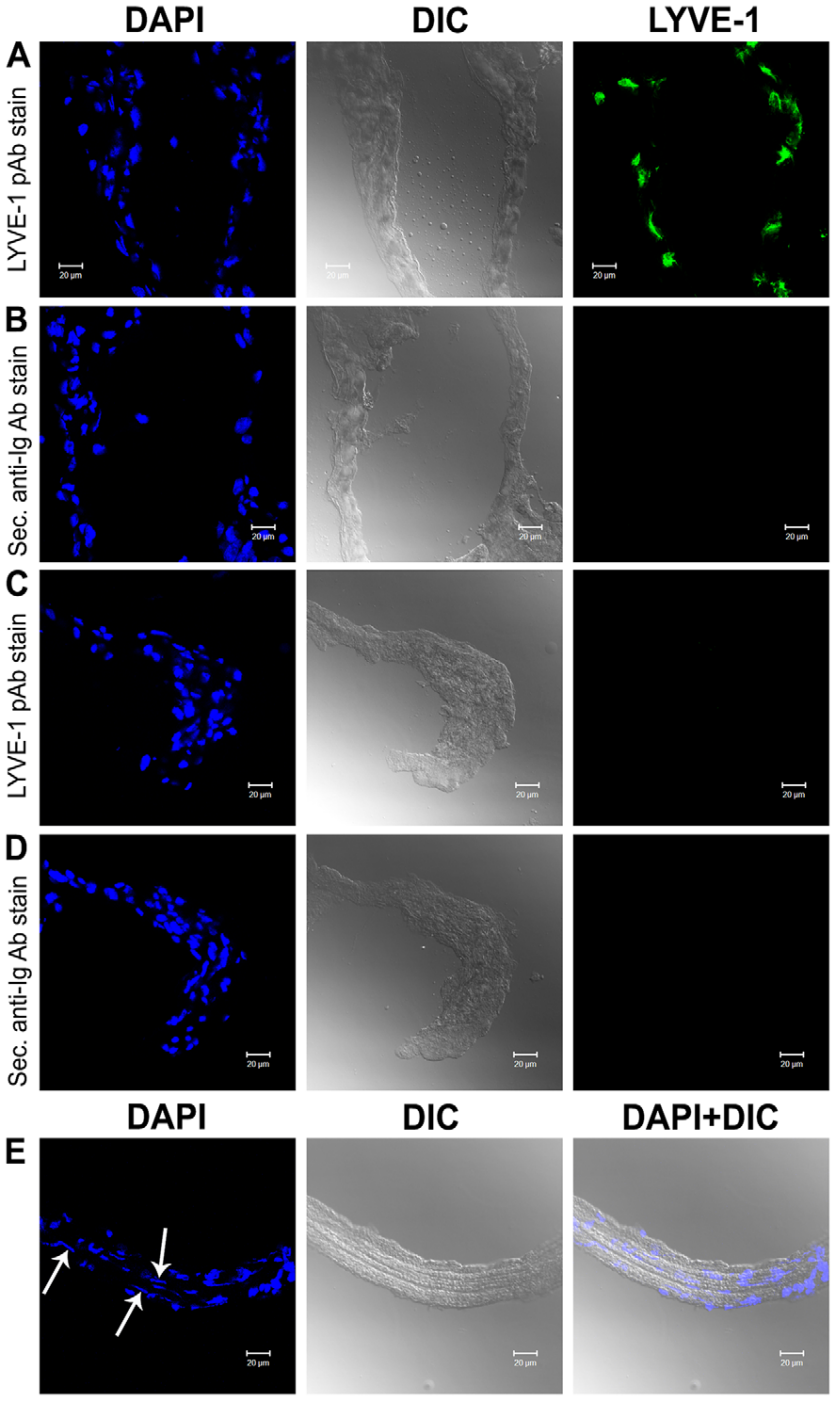
Immunofluorescence staining of the primo-vessel in a lung cancer xenograft. Primo-vessel cross sections were treated with anti-LYVE1 polyclonal antibody (green) or iso-type control polyclonal antibody (C, D) and observed with confocal microscopy. Lymphatic vessel cross sections were stained with the same antibodies and observed as positive controls (A, B). Fluorescence images of DAPI (blue) exhibiting nuclei and DIC images were also taken. Lymphatic endothelial cells with strong positive staining clearly located at the inner boundary of lymphatic vessel, whereas no LYVE-1 positive cells were seen in the primo-vessel. A part of the same sample which was treated with only DAPI without sectioning and mounted longitudinally revealed aligned rod-shape nuclei surrounding lumens assumed to be endothelial cells of primo-vessel (E).

To convincingly discriminate primo-vessels from lymphatic vessels, we next conducted immunohistochemical examination with LYVE-1. Figure 4 depicts the results of immunofluorescence staining of the primo-vessel in a lung cancer xenograft. Primo-vessel and lymphatic vessel cross sections were treated with anti-LYVE1 polyclonal antibody (green) or isotype control polyclonal antibody and were observed with fluorescence confocal microscopy. Fluorescence images of DAPI (blue)-exhibiting nuclei and DIC images were also taken. The lymphatic endothelial cells with strong positive staining are clearly located at the inner boundary of the lymphatic vessel (Fig. 4A) whereas no LYVE-1 positive cells are seen in the primo-vessel (Fig. 4C). Isotype control antibody treatment showed no fluorescent signal, proving the staining results to be reliable. A part of the same sample that had been treated with only DAPI without sectioning and had been mounted longitudinally revealed aligned rod-shaped nuclei surrounding the lumens, which nuclei were assumed to be endothelial cells of the primo-vessel (Fig. 4E).

## Discussion

The present study is the first anatomical demonstration and immunofluorescence investigation of the primo-vascular system, a new fluid-conducting vascular network, in lung cancer mouse models. Largely distributed primo-vessels were observed on the surfaces of abdominal organs in conjunction with tumor growth. Primo-vessels could be discriminated from similar-looking lymphatic vessels 1) by their high *in vivo* affinity with staining with trypan blue, 2) by their histologic features, i.e., multi-lumen structure with loose collageneous matrices, and 3) by their immunochemical characteristic, i.e., negative expression of LYVE-1.

Preferential staining of primo-vessels by trypan blue under *in vivo* conditions was previously reported [4]. In this article, we applied and removed 0.1% trypan blue under air, preventing generalized spread throughout the abdominal cavity, to reveal the entire distribution of primo-vessels with high contrast to background tissues. This high infiltration and staining of trypan blue on primo-vessels may be explained by the primo-vessels having more loose tissue matrix with some openings and pores at the outer boundary than other tissues. Trypan blue is a well-known vital stain and has been used to visualize the corneal endothelium in anterior segment surgery [17] or the anterior capsule in cataract surgery [18] or epiretinal membranes in proliferative vitreoretinopathy surgery [11]. This work confirmed the efficacy of using vital staining with trypan blue to visualize the distribution of abdominal primo-vessels.

We found well-developed abdominal networks of primo-vessels in tumor-bearing mice for the first time. Especially, we often observed primo-vessels connected to tumor nodules, with the growth of this novel architecture probably driven by the needs of the tumor, i.e. providing for nutritional supply to the tumor and for efficient transport of detached tumor cells for metastasis. Although further quantitative studies on tumor burden and morphometry of primo-vessels should be addressed, these preliminary anatomical findings may provide important implications for cancer prognosis and for therapy.

Because of the apparent morphological similarity - transparent thin structure - between lymph vessels and primo-vessels, distinguishing between them is critical. A distinction based on gross anatomical features [19] and histological details [12] in rabbits was reported before. The primo-vessels floating inside the flow of lymph vessels of rats and rabbits manifestly demonstrated that they are two different systems [20], [21], [22]. In this research, we accomplished not only a conventional histological but also immunofluorescence examination of primo-vessels obtained from tumor-bearing mice to verify the findings on previous reports. H&E and MT staining revealed multiple lumens surrounded with loose collagen fibers in primo-vessels, and those multi-lumen structures were distinct from the single-lumen structure of lymph or blood vessels. This result shows that primo-vessels cannot be mistaken for lymphatic vessels, coagulated fibrins, or other artifacts. Furthermore, a specific marker of lymphatics, LYVE-1, was not expressed at all in primo-vessels providing a clear difference between primo-vessels and lymph vessels. Rod-shaped nuclei aligned longitudinally around the lumens of primo-vessels, and although these nuclei were assumed to belong to endothelial cells, they were not lymphatic endothelial cells.

In the present work we focused on the difference between the primo-vessels and the lymphatic vessels because of their similarity in apparent looking. We did not emphasize the distinctive nature of the primo-vessels from the blood vessels because these two are manifestly discernible in the anatomical examination. Primo-vessels in the abdominal cavity are multi-lumen structure and do not adhere to the peritonea of internal organs (Figs. 1 and 2). In contrast, blood vessels are single-lumen structure and lie in the peritonea. Primo-vessels are semi-transparent, milky white colored and stained well with trypan blue under *in vivo* conditions while blood vessels are red colored and barely stained with trypan blue. Since the primo-vascular system is an entirely different system from the cardio-vascular system its formation should be a distinctive process from angiogenesis. It will be worthwhile testing this by using immunofluorescence with anti-CD31 antibody which is widely used for revealing endothelial cells of blood vessel origin.

Primo-vessels are often located along the exterior of blood vessels, so there might be a strong correlation between the densities of micro vessels and primo-vessels. At the present moment we can only mention that there is an indication that micro vessel density and primo-vessel density have a positive correlation in conjunction with tumor growth and metastasis. Our research is not the first to report new conduits which are distinctively different from blood or lymph vessels related to cancer events. Hendrix et al. found extracellular matrix-rich networks inside aggressive tumor tissue in 1999 [23]. They formed by a reversion of tumor cells to an undifferentiated phenotype and recapitulated embryonic vasculogenesis, the so-called vasculogenic mimicry [15]. In a similar way primo-vessels might be formed by embryonic vasculogenesis-like processes. Since primo-vessels were developed on the fascia of subcutaneous tissue in tumor bearing mice [6], it is an important issue to clarify the possible connection or relation of the vasculogenic-like networks and the primo-vessels between the inside and the outside tumor tissue.

In conclusion, we have demonstrated the extensive presence of primo-vessels in tumor-bearing mice and the prominent difference between the histological and the immunochemical features of primo-vessels and those of lymph vessels. These findings and analyses, which indicate a novel function of the primo-vascular system, different from that of lymphatics, in conjunction with cancer events, may provide the basis for a new therapy to control cancer growth and dissemination.

## Materials and Methods

### Cell Culture

NCI-H460 human lung cancer cells were obtained from the Korean Cell Line Bank (Seoul, Korea). The cells were cultured in a RPMI-1640 medium (Gibco-Invitrogen, USA) supplemented with 1% penicillin-streptomcyin and 10% fetal bovine serum (FBS) in 95% air and 5% CO_2_ at 37°C.

### Animal Cancer Model

Female nude mice (BALB-c-nu/nu, aged 5–7 weeks old, weighing 15–20 g, n = 20; Japan SLC, Inc., Hamamatsu, Japan) were used in accordance with institutional guidelines under approved protocols. Two sets of cancer models were used. The first model is the standard subcutaneous (s.c.) xenograft mouse model [9]. Ten mice were inoculated s.c. in the dorsum with 1×10^7^ NCI-H460 human lung cancer cells (in a 1-mL RPMI-1640 medium) for tumor formation under the skin. The other tumor model is the i.p. experimental metastasis model. NCI-H460 human lung cancer cells were injected i.p. as a cell suspension into nude mice (1×10^7^ cells per animals, n = 10) as previously described [9], [10]. All research involving animals have been approved by the Instisute of Laboratory Animal Resources of Seoul National University.

### Intraoperative Imaging

To identify primo-vascular system inside abdominal cavity of s.c. and i.p. lung cancer models, we performed mouse surgery two to eight weeks after inoculation of NCI-H460 cells under general anethesis with i.p. injection of zoletil/rompun. First, the midline of the mouse abdomen was incised and the intra-abdominal organs were exposed carefully. Then, a 0.1% trypan blue solution, previously filtered through 0.22-µm pore-sized filter paper, was applied drop wise on the exposed organs [4]. After rinsing away the dye with warm saline, primo-nodes and primo-vessels were identified by direct visualization of the surgical anatomy with a surgical microscope (SZX12, Olympus, Japan). Finally, the images were captured with a CCD camera (DP 70, Olympus, Japan).

### Histological Analyses

After intraoperative imaging of the primo-vascular system, *ex vivo* examination was performed with histological staining of the samples. For this purpose, primo-nodes and vessels were fixed in 10% Neutral Buffered Formalin (NBF) for 12 hours at 4°C, rinsed in phosphate buffered saline (PBS), dehydrated in a graded ethanol series, clarified in xylene, and embedded in Paraplast (Sigma, USA). Paraplast transverse sections, 6µm in thickness, being cut with a microtome were next stained with hematoxylin and eosin (H&E) for general morphological observation, and with Masson's trichrome (MT) to visualize the collagen fibers. Then the sections were mounted with Neomount, and examined with a light microscope (BX51, Olympus, Japan). Images were acquired with a CCD camera (Axiophot, Zeiss, Germany).

### Immunohistochemistry

To characterize primo-vessels against lymphatic vessels, we performed immunohistochemical investigation with the IgG anti-LYVE-1 polyclonal antibody (pAb), a specific marker of lymphatics. Primo-vessels and lymphatic vessels on the surface of abdominal organ in tumor bearing mice were harvested, embedded in OCT, and snap-frozen in liquid nitrogen. Cross-sections (10 µm) were fixed in ice-cold acetone for 5 min, washed in PBS, and blocked with CAS-Block solution (Invitrogen, USA) for 2h at 37°C. Slides were incubated with rabbit anti-mouse LYVE-1 (2.5 µg/ml, Abcam, UK) overnight at 4°C, followed by washes and detection with goat-anti rabbit IgG conjugated to Alexa Fluor 488 (1∶500, Molecular Probes, USA). Slides were treated with DAPI for nuclear counterstain, coverslipped in antifade reagent (Molecular Probes, USA). A part of the same sample was treated with only DAPI and longitudinally whole-mounted in antifade reagent. Samples were visualized by confocal microscopy (LSM500, Zeiss, Germany).
